# ASC-J9® increases the bladder cancer chemotherapy efficacy via altering the androgen receptor (AR) and NF-κB survival signals

**DOI:** 10.1186/s13046-019-1258-0

**Published:** 2019-06-24

**Authors:** Chi-Ping Huang, Jinbo Chen, Chi-Cheng Chen, Guodong Liu, Yong Zhang, Edward Messing, Shuyuan Yeh, Chawnshang Chang

**Affiliations:** 1 Sex Hormone Research Center and Department of Urology, China Medical University/Hospital, Taichung, 404 Taiwan; 2 Department of Urology, Xiangya Hospital, Central South University, Changsha, 410008 China; 3 George Whipple Lab for Cancer Research, Departments of Pathology, Urology, Radiation Oncology and The Wilmot Cancer Institute, University of Rochester Medical Center, Rochester, NY USA; 4 Department of Urology, Taichung Tzu Chi Hospital, Buddhist Tzu Chi Medical Foundation, Taichung, 404 Taiwan; 5 Department of Urology, the Second Hospital of Hebei Medical University, Shijiazhuang, 050000 China

**Keywords:** Bladder cancer, Androgen receptor, NF-κB, ASC-J9®, Cisplatin

## Abstract

**Background:**

The current chemotherapy regimens may extend survival for patients with metastatic bladder cancer (BCa) for a few months, but eventually most patients succumb to disease because they develop resistance to their chemotherapy.

**Methods:**

TCGA human clinical sample survey and urothelial tumor tissue microarrays (TMAs) were applied to investigate the expression of androgen receptor (AR) and NF-κB. Multiple BCa cell lines were used to test chemotherapy’s efficacy via multiple assays including XTT, flow cytometry, TUNEL, and BrdU incorporation. The effects of the AR degradation enhancer, ASC-J9®, combined with various chemotherapy reagents were examined both in vivo and in vitro.

**Results:**

We unexpectedly found that in muscle-invasive BCa (miBCa) the signals of both the AR and NF-κB were increased via a TCGA sample survey. Results from multiple approaches revealed that targeting these two increased signals by combining various chemotherapeutic agents, including Cisplatin, Doxorubicin or Mitomycin C, with ASC-J9® led to increase the therapeutic efficacy. The combined therapy increases the expression of the pro-apoptosis BAX gene and cell cycle inhibitor p21 gene, yet suppresses the expression of the pro-survival BCL2 gene in miBCa cells. Preclinical studies using an in vivo mouse model with xenografted miBCa cells confirmed in vitro cell line data showing that treatment with ASC-J9® combined with Cisplatin can result in suppressing miBCa progression better than Cisplatin alone.

**Conclusions:**

Together, these results support a novel therapeutic approach via combining Cisplatin with ASC-J9® to better suppress the progression of miBCa.

**Electronic supplementary material:**

The online version of this article (10.1186/s13046-019-1258-0) contains supplementary material, which is available to authorized users.

## Background

It has been projected that there will be 79,030 new bladder cancer (BCa) cases and 16,870 BCa deaths in the United States in 2017 [1]. BCa is the 4th most common newly diagnosed cancer and 8th leading cause of cancer-related deaths among males. However, it is not among the top 10 cancers among females [1]. Urothelial carcinoma is the most frequently diagnosed malignancy of the urinary bladder, comprising more than 90% of all bladder neoplasms [2, 3]. Approximately 25% of BCa patients are diagnosed with muscle-invasive disease (miBCa), while only a small proportion of BCa patients have distant metastases detectable at their initial diagnosis [2]. However, even with aggressive treatment, almost half of miBCa patients eventually develop clinically apparent distant metastases [2].

The primary treatment for metastatic bladder cancer (MBC) is systemic chemotherapy and the standard of care is to use these same chemotherapy regimens, along with or without local treatment (eg. surgery or radiation therapy) to treat patients with miBCa [4]. While most effective chemotherapy regimens, gencitabine/cisplatin (GC) and methotrexate/vinblastine/doxorubicin/Cisplatin (MVAC), for MBC and miBCa all include Cisplatin, yet most patients with MBC ultimately die of their malignancy. Therefore, how to improve the efficacy of this chemotherapy with Cisplatin is crucial to obtain better outcomes in this disease [5].

Tumor heterogeneity and acquired resistance in BCa cells with higher mutation frequencies may contribute to chemotherapy’s failure and resistance to targeted therapy [6, 7]. The molecular profiling of miBCa from the TCGA database has provided valuable information about the genetic alterations in miBCa [8].

ASC-J9® (l,7-Bis-(3,4-dimethoxy-phenyl)-5-hydroxy-hepta-l,4,6-trien-3-one), a recently developed enhancer of androgen receptor (AR) degradation, has been shown to suppress prostate, bladder, liver, and kidney cancers in both in vitro cell lines and in vivo mouse models via targeting the AR and/or other mechanisms [9–11]. Its ability to increase chemotherapy efficacy in miBCa, however, remains unclear. Here we found ASC-J9® with Cisplatin can increase chemotherapy’s efficacy to suppress miBCa progression.

## Materials and methods

### Cell culture and reagents

Human BCa J82 (AR-negative) and TCC-SUP (AR-positive) cells were obtained from the American Type Culture Collection (ATCC) in August 2015 and maintained in DMEM supplemented with 10% fetal bovine serum. The cells were characterized by ATCC using DNA profiling (short tandem repeat), cytogenetics, and isoenzyme analysis and were used from replicate frozen stocks derived within 6 months of receipt. ASC-J9® was a gift from AndroScience. The chemical structure of ASC-J9® was described previously [12]. Cisplatin was purchased from Sigma Co. Doxorubicin and mitomycin C were obtained from China Medical University Hospital (CMUH) pharmacy. The Cisplatin-resistant (Cis-R) BCa cell lines were established by stepwise increments of exposure to Cisplatin, starting with 0.02 μM Cisplatin for 4 weeks and with 0.2 μM Cisplatin for another 4 weeks and finally with 2 μM Cisplatin for another 4 weeks to become more resistant to Cisplatin treatment than parental cells.

### Tissue microarrays (TMAs)

TMAs were generated using urothelial tumor tissue cores from a cohort of patients treated at the CMUH, Taichung, Taiwan by searching the Institution’s pathology database. The tumors originated in the renal pelvis and ureter, as well as in the urinary bladder. TMAs were constructed using a semi-automated arraying device (TMArrayer™, Pathology Devices, Westminster, MD, USA). One to three tissue cores (each 2 mm) of representative areas from each of the selected formalin-fixed, paraffin-embedded tissue blocks were used for the array. Immunohistochemistry was performed on the TMA with a rabbit anti-nuclear factor-kappa B (NF-κB) p65 antibody using a Leica BondIII autostainer (Leica Microsystems, Mount Waverley, Victoria, Australia) according to the manufacturer’s protocol. Slides were then counterstained with hematoxylin.

### Transient transfection and promoter reporter assay

J82 and TCC-SUP cells were seeded onto 12-well plates and transfected with 0.5 μg pGL plasmid containing NF-κB response element (RE) using Lipofectamine (Invitrogen, Carlsbad, USA). To monitor the transfection efficiency, a pTK Renilla luciferase (RL) plasmid (50 ng) encoding RL was included in all transfections. At 24 h post-transfection, the cells were treated with ASC-J9®. After another 24 h, the cell lysates were collected and the levels of firefly and RL activity were measured sequentially by a luminometer from a single sample using the Dual-Glo Luciferase Assay System (Promega, Madison, WI USA). RL activity was used as an internal control to normalize firefly luciferase activity.

### Total and phosphorylated NF-κB p65 assay

J82 and TCC-SUP cells were treated with ASC-J9® or vehicle control. After 24 h, the cell lysates were collected and analyzed for total and phosphorylated NF-κB p65 by InstantOne™ ELISA (eBioscience, San Diego, CA, USA), following the manufacturer’s protocol.

### XTT cell viability assay

To test the effects of the agents, the cells were seeded at 2 × 10^4^ cells/well onto 96-well plates and then incubated overnight. At time 0, media were replaced with fresh complete media vehicle control, ASC-J9®, Cisplatin, Doxorubicin, Mitomycin C, or combinations, which were added at concentrations as indicated. After 48 h, an XTT assay kit (Sigma-Aldrich, St. Louis, MO, USA) was used to measure cell viability, which was expressed as a percentage of the absorbance measured in the vehicle treated cells. The IC50 value of each treatment was calculated based on the dose-response curves produced by the XTT assays. In order to determine whether the combined treatment was synergistic or additive, the data on cell viability for each treatment alone and for the combination treatment with ASC-J9® were analyzed with Calcusyn software (Biosoft, Cambridge, UK) to determine combination index (CI).

### Cell cycle and apoptosis detection by flow cytometry

Cells were harvested after treatment and resuspended in 200 μl PBS containing 1 μg/ml DAPI for 30 min at room temperature in the dark. The DNA contents of stained cells were analyzed using a flow cytometry system. The distribution of cells in the cell cycle and the percentage of cells below the G1 peak (subG1 fraction, apoptotic cells) were analyzed using FlowJO software.

### In situ DNA fragmentation assay (TUNEL assay)

To examine the effect of treating J82 and TCC-SUP cells with Cisplatin, ASC-J9®, and the combination on cell apoptosis, apoptotic cells were analyzed by In Situ DNA Fragmentation Assay Kit by labeling DNA breaks as described by the manufacturer (Biovision, Mountain View, USA). Briefly, the cells were seeded at 5 × 10^5^ cells on cover slips and cultured for 1 to 3 days to allow the growth of a nearly confluent monolayer and treated with Cisplatin, ASC-J9® or the combination.

### Bromodeoxyuridine (BrdU) incorporation assay

To examine the effect of treating J82 and TCC-SUP cells with Cisplatin, ASC-J9® and the combination on the cell proliferation, we analyzed cells proliferation by the BrdU incorporation assay using BrdU In-Situ Detection as described by the manufacturer (BD Biosciences, San Jose, CA, USA).

### Western blotting

Protein lysates were separated by 10% SDS-PAGE and then transferred to nitrocellulose membranes by electroblotting. The membranes were incubated with anti-IκB kinase, anti-GADPH, anti-BCL2, anti-BAX, anti-p21, or anti-β-ACTIN antibodies (Cell Signaling Technology, Beverly, MA, USA) overnight at 4 °C before subsequent incubation with secondary antibodies conjugated with horseradish peroxidase for 1 h at 37 °C. Proteins were visualized using enhanced chemiluminescence reagent.

### Quantitative real-time PCR for RNA analysis

The expressions of BCL2, BAX, and p21, relative to the housekeeping gene β-ACTIN, in BCa cells were measured by real-time PCR. Total RNAs were extracted from cells using TRIzol (Invitrogen, Carlsbad, CA, USA) and used for first-strand cDNA synthesis. The mRNA levels were measured by CFX96™ real-time system (Bio-Rad Laboratories) using KAPA SYBR® fast qPCR kits (Kapa Biosystems, Inc., Woburn, MA, USA). The mRNA expression levels were determined using the 2^-(∆∆Ct)^ method.

### BCa cell xenografts in nude mice and treatment regimen

Male BALB/c nude mice aged 5 to 6 weeks were purchased from the National Laboratory Animal Center (NLAC) (Taiwan). Animal handling and experimental procedures were approved by the Animal Experiments Ethics Committee of the China Medical University. To measure tumor growth affected by ASC-J9® and anti-tumor drugs, mice were injected subcutaneously into the posterior flank (5 mice per group) with J82 cells (1 × 10^6^ cells in 100 μl, at 1:1 with matrigel). After tumors developed to ~ 200 mm^3^ for systemic administration, mice were treated with intraperitoneal (i.p.) injection of vehicle, 2.5 mg/kg Cisplatin every week, 50 mg/kg ASC-J9® 3 times a week, or both drugs, for a total of 4 weeks. Tumor volumes were measured 3 times per week and calculated according to the following formula: Volume = (A × B^2^)/2, where A is the largest diameter and B is the shortest diameter. Fractional tumor volume was used to evaluate treatment efficacy. The fractional volumes were calculated as the fold increase over the original (day 0 of treatment) tumor volume and graphed as fold increase in volume ± SD for each treatment. After 4 weeks of treatment, the mice were sacrificed and the tumors were fixed for IHC staining.

### Immunostaining for apoptosis, PCNA, BCL2, BAX, and phospho NF-κB p65

Formalin-fixed, paraffin-embedded tissue sections (4 μm) were deparaffinized, rehydrated, and washed in PBS. Endogenous peroxidase was quenched. Apoptosis was measured by TUNEL assay using a commercially available apoptosis In Situ detection kit. To determine cell proliferation, cell apoptosis and NF-κB activation, PCNA, BCL-2, BAX, and phospho-NF-κB p65 immunostainings were performed using the PCNA, BCL-2, BAX, and phospho-NF-κB p65 antibodies, respectively. Visualizations of the immunoreactions were performed with 0.06% 3,3′-diaminobenzidine (DAB). IHC results were scored by P x I (multiplying the percentage of positive cells (P%) by the intensity (I), range from 1: weak staining; 2: moderate staining to 3: strong staining. Maximum = 300.

### Statistical analysis

The results for each treatment group were presented as a mean of at least 3 experiments with each data point performed in triplicate. The mean values and standard errors/deviations were calculated for each treatment group from the pooled normalized data. The statistical significance of the difference between groups was determined by the two-tailed Student’s t-test. Values of *p* < 0.05 were considered significant.

## Results

### Increased expression of NF-κB and AR in miBCa

**Fig. 1:**
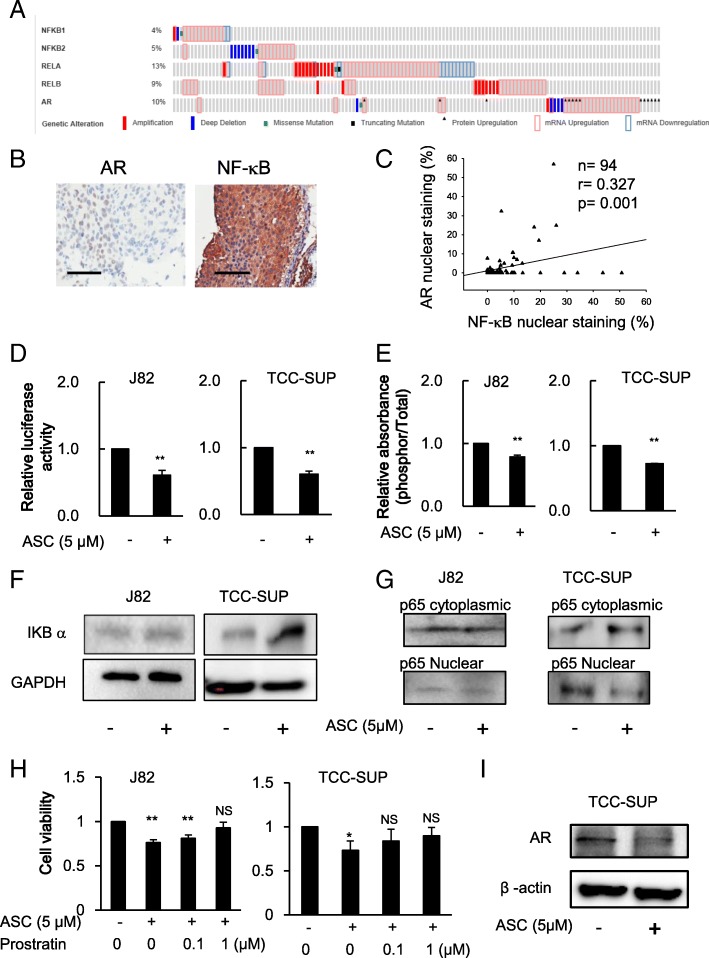
Higher NF-kB and AR expression correlates with muscle-invasive BCa in human clinical samples and ASC-J9® treatment repressed NF-κB signaling pathway in J82 and TCC-SUP cells and decreased AR expression in AR positive TCC-SUP cells. **a** Oncoprints of NFKB1, NFKB2, RELA, RELB, and AR genomic alterations. Individual genes are represented in each row, and individual tumors are represented as columns. Genetic alterations are color coded: dark blue, homozygous deletion; red, amplification; green, misssense mutation; black, truncating mutation; black arrowhead, protein upregulation; pink frame, mRNA upregulation; and light blue frame, mRNA downregulation. The oncoprints are based on data obtained from the cBioPortal for Cancer Genomics (http://www.cbioportal.org/). **b** Representative images of AR and NF-kB p65 staining in muscle invasive urothelial tumor tissues. Scale bar, 100 mm. **c** The Pearson correlation (shown as key) between AR and NF-kB nuclear expression. **d** NF-κB transactivation affected by ASC-J9® (ASC) was determined using reporter gene assay with plasmids containing luciferase gene under NF-κB response element (NRE) control in J82 and TCC-SUP cells after 24 h treatment. **e** The activation of NF-κB affected by ASC was measured by ELISA to determine the phospho-p65 in J82 and TCC-SUP cells after 24 h treatment. **f** The protein expression of IκBα affected by ASC-J9® was measured using western blotting after 48 h treatment in J82 and TCC-SUP cells. **g** Decreased levels of p65 protein in the nuclear fraction of J82 and TCC-SUP cells following ASC-J9® treatment. **h** The effect of IκB kinase (IKK) activator, prostratin, on the suppressive activity of ASC on cell growth was determined in J82 and TCC-SUP cells after 48 h treatment. **i** ASC-J9® decreased AR protein levels in TCC-SUP cells after 48 h treatment. Data are presented as mean ± SD; *, *p* < 0.05; **, *p* < 0.01, vs no treatment control, NS = Not Significant

To identify potential therapeutic targets to improve suppression of miBCa, we applied the cBioPortal to analyze the TCGA (The Cancer Genome Atlas) BCa database to search for altered signals that might be linked to BCa progression [13, 14]. The results revealed that higher expression of NF-κB (NFKB1, NFKB2, RELA and RELB) and AR in BCa occurred in nearly 37% (47 of 126) of cases (Fig. 1a). To further verify these human clinical survey data, we then performed immunohistochemistry (IHC) staining of AR and NF-κB signals in the collected urothelial tumors from 90 patients with miBCa or upper urinary tract tumors. The results (Fig. 1b-c) revealed that nuclear staining of the AR is positively correlated with nuclear location of NF-κB (p65, Rel A). Because prior studies indicated that NF-κB and AR signals might play important roles in BCa’s progression [15, 16], we were interested in seeing if targeting these increased NF-κB and AR signals may improve the efficacy of chemotherapy to suppress miBCa.

### ASC-J9® suppresses AR expression and NF-κB activity in miBCa cells

Recent studies indicated that ASC-J9® could suppress BCa growth via degrading AR as well as via other mechanisms [9, 17]. Interestingly, as a derivative of Curcumin, ASC-J9® may also have anti-NF-κB activity that could suppress the growth of different tumors [18–20]. We therefore hypothesized that ASC-J9® might be able to suppress miBCa progression via targeting both AR and NF-κB signals.

We first found that ASC-J9® could suppress the transcriptional activation of NF-κB via a promoter gene luciferase assay in the human miBCa J82 and TCC-SUP cells (Fig. 1d). Results from the measurement of the ratio of total and phosphorylated (active) human NF-κB p65 (phospho-Ser536, that is phosphorylated by IκB kinases) [21, 22] also confirmed that activation of NF-κB was suppressed by ASC-J9® treatment in both cell lines (Fig. 1e). We then measured whether ASC-J9® could suppress IκB kinase activity and result in increased IκB protein levels, since NF-κB is activated by moving from the cytoplasm to the nucleus upon phosphorylation/degradation of its inhibitory molecule IκB. The results revealed that ASC-J9® was able to increase IκB protein in both J82 and TCC-SUP cells (Fig. 1f). Furthermore, to directly assay NF-κB activation by determining NF-κB p65 nuclear and cytoplasmic fractions, we used western blot analysis and detected decreased translocation of p65 into the nuclear fractions in the presence of ASC-J9® (Fig. 1g) compared to the cytoplasmic fractions. Importantly, treating with prostratin, an IκB kinase (IKK) activator [23], could then lead to reverse the suppressive effect of ASC-J9® on cell growth in J82 and TCC-SUP cells (Fig. 1h), suggesting that the regulation of IκB kinase activity is involved in the effect of ASC-J9® on cell growth. As expected, we also found that ASC-J9® could decrease AR protein in AR positive miBCa TCCSUP cells (Fig. 1i).

Together, results from Fig. 1d-i demonstrated that ASC-J9® could suppress both NF-κB and AR signals in miBCa cells.

### ASC-J9® increases the chemotherapy efficacy with Cisplatin, Doxorubicin or Mitomycin C to better suppress miBCa cell growth

Chemotherapy with Cisplatin and Doxorubicin has been used to treat miBCa patients [4]. Mechanistic studies suggest that Cisplatin, a platinum-containing anti-cancer drug, can cross-link to DNA to trigger apoptosis and cell cycle arrest [24, 25]. Doxorubicin is an anthracycline antibiotic that functions through intercalating into DNA to inhibit topoisomerase II, thereby stopping DNA replication (25). Mitomycin C, the chemotherapeutic agent to treat non-muscle-invasive BCa (nmiBC) can induce reactive oxidative species (ROS) to promote cell apoptosis and function as a potent DNA cross-linker to alter DNA alkylation and cause cell cycle arrest [26].

These findings/reports suggest these anti-BCa chemotherapy agents may function by enhancing apoptosis and/or inducing cell cycle arrest suppressing tumor progression [27]. However, most patients with MBC who received these chemotherapies eventually develop resistance after an average of 7 months treatment [28].

**Fig. 2:**
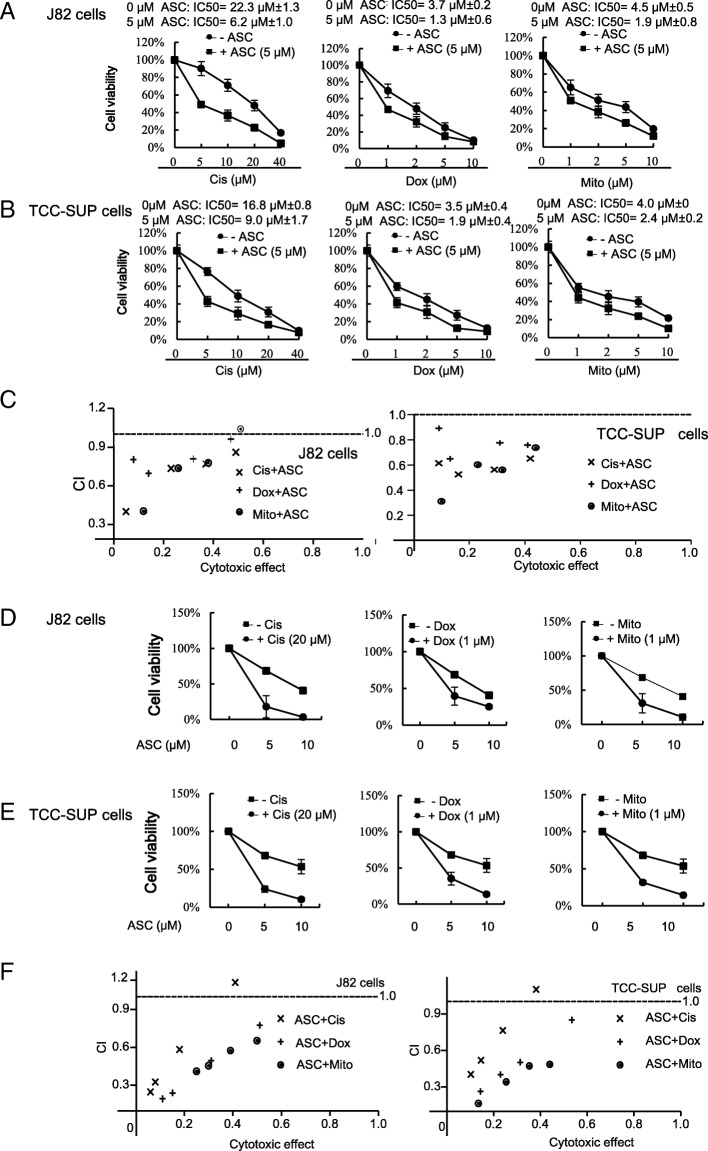
ASC-J9® sensitizes bladder cancer cells to Cisplatin, Doxorubicin or Mitomycin C anti-growth activity. **a**-**b** BCa cells were plated in 96-well plates to adhere overnight. The following day, the plates were rinsed and 10% serum containing media with designated doses of ASC-J9® (ASC), Cisplatin (Cis), Doxorubicin (Dox), or Mitomycin C (Mito) were added. After 48 h, relative viable cell numbers of J82 (**a**) and TCC-SUP (**b**) cells after treating were determined using a XTT assay. Cell numbers were standardized to cells incubated without ASC. **c** The combination index (CI) of Cis, Dox, or Mito in combined treatment with ASC in J82 or TCC-SUP cells. **d**-**e** The relative viability of J82 (**d**) and TCC-SUP (**e**) cells to different doses of ASC with Cis, Dox, or Mito treatment. **f** The combination index (CI) of ASC treatment in combination with Cis, Dox or Mito in J82 or TCC-SUP cells. For (**c**) and (**f**), CI = < 1, indicates synergism; CI = 1 indicates an additive effect; and CI = > 1 indicates drug antagonism. Data are presented as mean ± SD

We first treated miBCa J82 cells with Cisplatin, Doxorubicin, or Mitomycin C, alone or combined with ASC-J9® and examined BCa cell growth via XTT assays [29]. Results revealed that the IC50 inhibition of cell growth for these chemotherapy agents was decreased after being combined with 5 μM ASC-J9® (Fig. 2a). Similar results were obtained with another human miBCa cell line, TCC-SUP, showing that IC50 inhibition of cell growth decreased when combined with ASC-J9® (Fig. 2b).

Importantly, we found a synergistic cytotoxicity when ASC-J9® was combined with different concentrations of Cisplatin, Doxorubicin, and Mitomycin C to treat both J82 and TCC-SUP cells (CI < 1) (Fig. 2c), and ASC-J9® alone could also suppress cell growth in a dose-dependent manner in both J82 cells (Fig. 2d) and TCC-SUP cells (Fig. 2e). As expected, a synergistic cytotoxicity (CI < 1) was also observed when Cisplatin, Doxorubicin, and Mitomycin C were combined with different concentrations of ASC-J9® to treat both J82 and TCC-SUP cells (Fig. 2f).

Together, results from Fig. 2a-f indicated that treating with ASC-J9® could increase the chemotherapy efficacy of Cisplatin, Doxorubicin, and Mitomycin C. We then chose Cisplatin for further studies since it is the most often used drug in the current chemotherapy for MBC and miBCa [4].

### ASC-J9® can still suppress miBCa cells that already developed chemo-resistance to Cisplatin

**Fig. 3:**
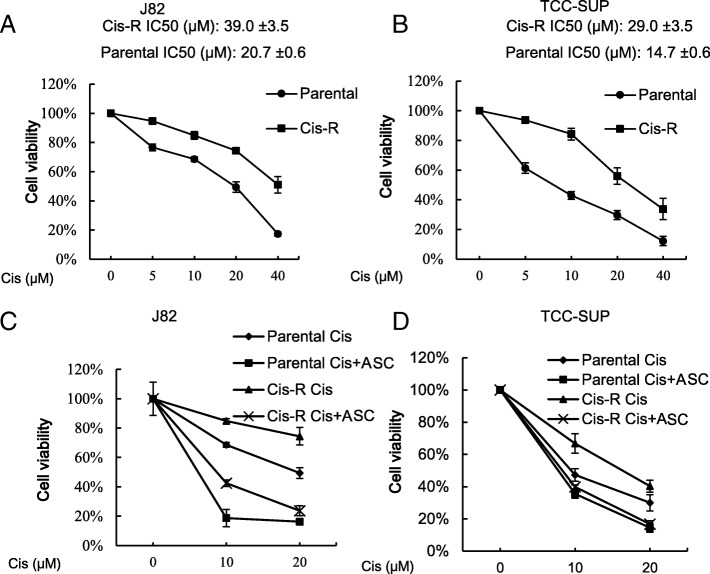
ASC-J9® (ASC) alone or in combination with Cisplatin (Cis) decreased the growth of cisplatin resistant (Cis-R) BCa cells compared to parental Cis sensitive cells. **a**-**b** XTT assay demonstrates the viability of Cis-R J82 (**a**) and TCC-SUP (**b**) cells developed by sequential Cis exposures. **c**-**d** XTT assay of the Cis-R J82 (**c**) and TCC-SUP (**d**) cells treated with Cis, ASC or both. Data are presented as mean ± SD

In addition to increasing chemotherapy’s efficacy, we were interested to see if ASC-J9® treatment could suppress the growth of miBCa cells that had already developed Cisplatin-resistance. We first established the Cisplatin resistant (Cis-R) miBCa J82 and TCC-SUP cells by sequential Cisplatin exposures, and demonstrated that the IC50 of these Cis-R J82 (Fig. 3a) and TCC-SUP (Fig. 3b) cells was higher than the original parental Cisplatin-sensitive miBCa cells. Importantly, we found that treating these Cis-R J82 (Fig. 3c) and TCC-SUP (Fig. 3d) cells with ASC-J9® also led to a better cytotoxicity in combination with Cisplatin, indicating that ASC-J9® also has the capacity to suppress the miBCa cells that were developed to have Cisplatin chemo-resistance.

### Mechanism dissection of how ASC-J9® increases Cisplatin chemotherapy efficacy: via altering the miBCa cell apoptosis and proliferation

**Fig. 4:**
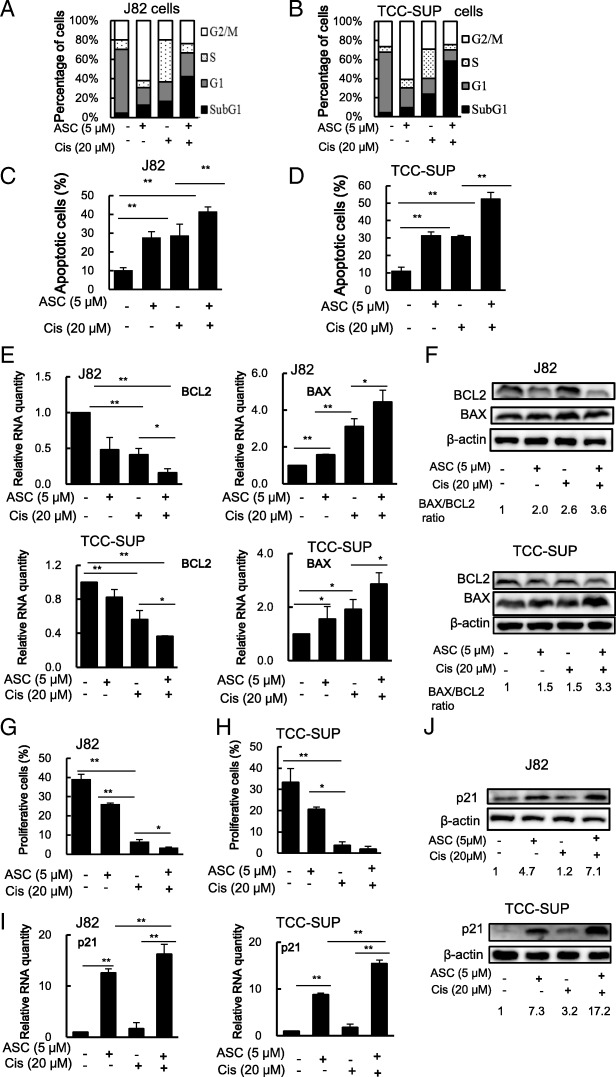
ASC-J9® (ASC) alters cell cycle profiles and apoptotic cell population of BCa cells treated with Cisplatin (Cis). **a**-**b** Cell cycle profile and apoptotic cell population in J82 (**a**) and TCC-SUP (**b**) cells treated with Cis with/without 5 μM ASC. Cell cycle and apoptosis analyses were assessed by 2 μg/ml DAPI staining for 10 min at room temperature followed by flow cytometry. Apoptotic cells were determined by evaluating the percentage of events accumulated in the subG1 position. **c**-**d** The Apoptosis TUNEL Assay was used to determine the apoptosis affected by Cis, ASC, or combined treatment in J82 (**c**) and TCC-SUP (**d**) cells. **e**-**f** Apoptosis signals of BCL-2 and BAX affected by treatment with Cis, ASC or both were evaluated by RT-PCR (**e**) and by Western blotting (**f**) assay. **g**-**h** The cell proliferation BrdU incorporation assay and cell proliferation regulatory proteins were affected by Cis, ASC or combined treatment in J82 (**g**) and TCC-SUP (**h**) cells. **i** Cell growth inhibitory signal p21 RNA affected by treatment with Cis, ASC or both on J82 and TCC-SUP cells were evaluated by RT-PCR. **j** Cell growth inhibitory signal of p21 proteins affected by treatment with Cis, ASC or both on J82 (upper) and TCC-SUP (lower) cells were evaluated by Western blotting assay. For (**f**) and (**j**) the relative p21 expressions (the number under the bands) were determined by measuring densities of corresponding bands on western blots and normalizing to β-ACTIN. Data are presented as mean ± SD. *, *p* < 0.05; **, *p* < 0.01

To dissect the mechanisms of how ASC-J9® increases Cisplatin chemotherapy efficacy, we treated J82 or TCC-Sup cells with 5 μM ASC-J9®, 20 μM Cisplatin, or combined Cisplatin with ASC-J9® for 48 h, and results from cell cycle analyses revealed that profiles (the percentages of cells in G1, S, and G2/M phases) of J82 cells were shifted from 66, 10 and 20%, to 18, 7 and 62% after 48 h ASC-J9® treatment, to 20, 43 and 20% after Cisplatin treatment, and to 24, 9, and 24%, after combining Cisplatin with ASC-J9® (Fig. 4a). The percentage of cells in G1, S, and G2/M phases of TCC-SUP cells were shifted from 63, 6 and 26%, to 21, 9 and 60% after 48 h ASC-J9® treatment, to 17, 31, and 29% after Cisplatin treatment, and to 12, 5 and 24% after combining Cisplatin with ASC-J9® (Fig. 4b). These changes indicated a better effect of ASC-J9® and Cisplatin combined treatment on cell cycle regulation.

Among these changes, we noticed that the percentage of J82 cells in the subG1 population, representing apoptotic cells, increased from 4 to 13% with ASC-J9® treatment, to 17% with Cisplatin treatment, and to 42% with combined treatment (Fig. 4a). The percentage of TCC-SUP cells in the subG1 population also increased from 4 to 9% with ASC-J9® treatment, to 23% with Cisplatin treatment, and to 58% with combined treatment (Fig. 4b), suggesting ASC-J9® may function mainly by enhancing apoptosis to increase Cisplatin chemotherapy efficacy to better suppress miBCa cell growth. Using TUNEL assay, we also confirmed that Cisplatin alone or ASC-J9® alone induced cell apoptosis, and combined treatment further increased apoptotic cell numbers (Fig. 4c&d & Additional file 2: Figure S2A-B).

Together, results (Fig. 4a-d) from cell cycle prolife and multiple apoptosis assays suggest that ASC-J9® may function mainly through altering the miBCa cell apoptosis to increase Cisplatin chemotherapy efficacy to better suppress miBCa cell growth.

To further confirm the potential impact of ASC-J9® on apoptotic related signals, we also assayed the apoptosis related genes expression, and results revealed ASC-J9® treatment could further decrease Cisplatin-suppressed BCL-2, an important molecular player in anti-apoptotic signals, which was directly transcriptionally regulated by NF-kB with a binding site in the BCL-2 p21 promoter [30], at both RNA and protein levels in both J82 and TCC-SUP cells (Fig. 4e-f). In contrast, ASC-J9® treatment could increase the Cisplatin-enhanced BAX, an important player in the pro-apoptotic signals, at both RNA and protein levels in both J82 and TCC-Sup cells (Fig. 4e-f).

Together, these results from Fig. 4a-f further prove that ASC-J9® can increase Cisplatin chemotherapy efficacy via altering miBCa cells’ apoptotic signals.

In addition to altering miBCa cells’ apoptosis, ASC-J9® also functions via decreasing BCa cell proliferation to increase Cisplatin-suppression of BCa cell growth. Results from the BrdU incorporation assay [31] revealed that Cisplatin or ASC-J9® treatment alone reduced the number of cells entering the cell cycle and the combined treatment had a much greater effect on both J82 and TCC-SUP cells (Fig. 4g-h). We also examined the expression of cell cycle regulatory protein p21, an inhibitor of cyclin-dependent kinases (CDKs) [32] that are downregulated by the AR [33], and results revealed that treating with ASC-J9® or Cisplatin could increase p21 RNA (Fig. 4i) and protein expression (Fig. 4j) in both J82 and TCC-SUP cells. The combined treatment could further increase the p21 RNA and protein levels, suggesting Cisplatin and ASC-J9® may also function via altering cell cycle network signals to suppress miBCa cell proliferation.

Together, results from Fig. 4g-j suggest that ASC-J9® can also increase Cisplatin chemotherapy efficacy via altering miBCa cells’ proliferation signals.

### Preclinical study using a mouse model to prove combining ASC-J9® with cisplatin enhances suppression of miBCa progression

**Fig. 5:**
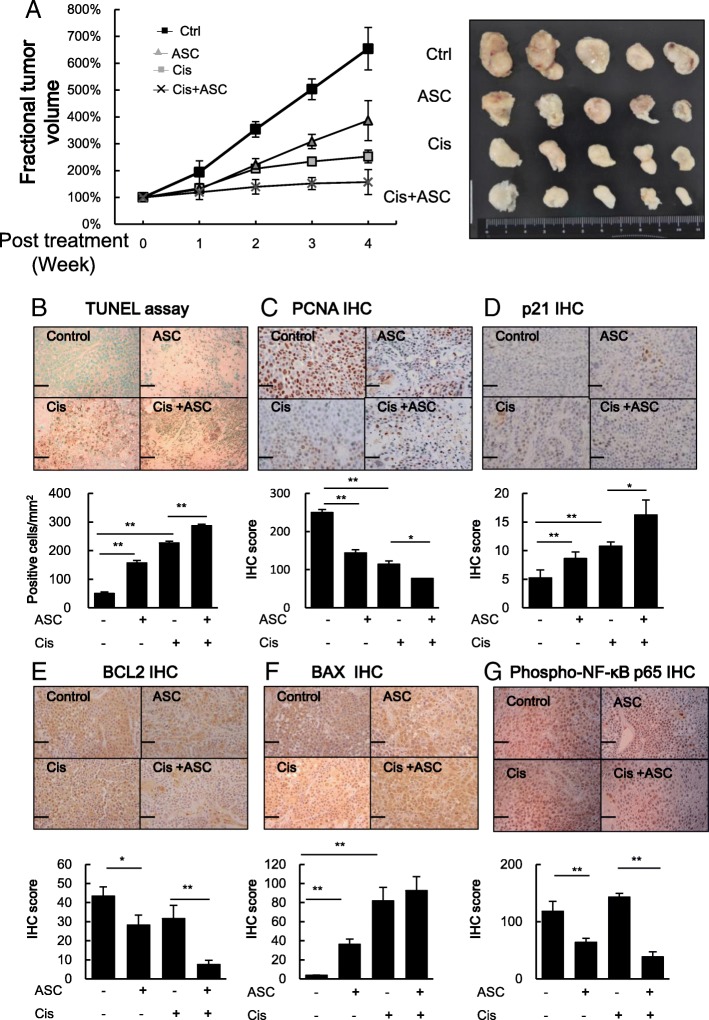
The effects of ASC-J9® (ASC) and Cisplatin (Cis) treatment in J82 xenograft tumors. Nude mice bearing ~ 200 mm^3^ J82 xenograft tumors were treated with vehicle control, ASC (50 mg/kg/mouse 3 times a week), Cis (2.5 mg/kg/mouse once a week) or both for 4 weeks. Tumor growth and volumes were monitored and measurements taken on the days indicated (*n* = 5 per group). **a** The fractional tumor volume curve (left panel) over several days of treatment. Tumor volumes were calculated by the following formula: volume = (length x width^2^)/2. For each tumor, fractional tumor volumes were calculated using the following formula: Fractional tumor volume = (volume on day measured)/(initial tumor volume). The right panel is the image of dissected tumors in each treatment group. **b**-**c** Representative images (upper panels) and quantification (lower panels) of (**b**) apoptotic cells in the xenograft tumors from each group as determined by TUNEL assay and (**c**) cell proliferation as determined by PCNA staining. **d**-**g** Representative images (upper panels) and quantification (lower panels) of immunostaining of cell cycle related gene p21 (**d**), apoptosis related genes BCL-2 (**e**) and BAX (**f**) and activated NF-κB (**g**), using antibodies against phospho-p65 in xenograft tumors. Scale bars, 50 μm. Data are presented as mean ± SD. *, *p* < 0.05; **, *p* < 0.01

Finally, to further confirm the above in vitro cell line results in the in vivo mouse model, we subcutaneously injected miBCa J82 cells into the right flank of nude mice and monitored tumor growth. When the tumors reached over 200 mm^3^, the mice were randomized into different treatment groups and then i.p. injected with vehicle control, ASC-J9® (50 mg/kg body weight/3 times per week), Cisplatin (2.5 mg/kg body weight once per week), or the same doses of ASC-J9® combined with Cisplatin for a total of 4 weeks (see the treatment scheme in Additional file 1: Figure S1). The tumor sizes were measured every 3 days for 4 weeks before sacrifice. As shown in Fig. 5a, ASC-J9® or Cisplatin treatment effectively reduced the growth rate of all xenografted tumors compared with control, and Cisplatin combined with ASC-J9® treatment led to the best suppressive effects on xenografted BCa tumor growth.

Results from the TUNEL assay further demonstrated that ASC-J9® or Cisplatin treatment increased apoptotic cell numbers and the combination further enhanced apoptosis (Fig. 5b). Furthermore, IHC staining on xenograft tumors with antibodies against proliferation marker PCNA revealed decreased BCa cell proliferation, with the fewest cell proliferation signals in the tumors with the combined treatment (Fig. 5c). IHC on xenograft tumors also revealed p21 protein expression was induced by ASC-J9® or Cisplatin treatment and the combination treatment had the highest induction (Fig. 5d). IHC on xenograft tumors with antibodies to apoptosis related genes including BCL-2 (Fig. 5e) and BAX (Fig. 5f) also confirmed Cisplatin with ASC-J9® treatment had the best suppressive effects on xenografted miBCa tumor growth via increased miBCa cell apoptosis.

We also examined NF-κB activation in xenografted tumors using immunostaining with antibodies against phospho-p65 (phospho-Ser529), which is phosphorylated by casein kinase II and enhances NF-κB transcriptional activity by increasing nuclear translocation of the activated complex and DNA binding properties [34]. Results (Fig. 5g) revealed that NF-κB activation was repressed most in the combination treatment group.

Together, in vivo results from Fig. 5a-g conclude that ASC-J9® can increase Cisplatin chemotherapy efficacy in miBCa via altering cell apoptosis and proliferation.

## Discussion

Treatment with Cisplatin chemotherapy has been the standard care for advanced BCa since the late 1980s [27]. However, due to impaired renal function and/or hearing, 24–52% of BCa patients are ineligible for Cisplatin treatment [35]. Therefore, our finding that combining Cisplatin therapy with ASC-J9® to increase Cisplatin’s efficacy may improve cisplatin’s ability to better treat BCa patients.

Cisplatin is administered intravenously in patients in doses ranging from 50 to 100 mg/m^2^ and the present pharmacokinetic and pharmacodynamic studies suggest that the Cmax or steady-state plasma level of Cisplatin should be maintained between 1.5 and 2 μg/ml (5 to 6.6 μM) in a standard continuous infusion schedule over 2 h and 4 h [36]. The IC50 of Cisplatin we tested on BCa cells ranged from 16 to 22 μM and was reduced to 6 to 9 μM by ASC-J9®, suggesting that adding ASC-J9® in the chemotherapy with Cisplatin (or Cisplatin containing regimens) could reduce the dose lethal to BCa, so that more patients may be eligible for Cisplatin chemotherapy, if future clinical trials proved ASC-J9® has little nephrotoxicity or toxicity. Currently, ASC-J9® has completed a phase 2B acne clinical study as a topical cream for treatment of acne vulgaris and it exhibits a positive treatment response and a safe drug profile (NCT01289574, https://clinicaltrials.gov/). However, its use as a systemic anti-tumor agent is still in preclinical development.

ASC-J9®, an AR degradation enhancer, can selectively target AR without influencing libido, fertility, and/or sexual behavior [37]. Importantly, an early study also indicated that ASC-J9® had therapeutic effects on spinal bulbar muscular atrophy (SBMA) in mice via degradation of AR mutants with little influence on serum testosterone levels [37]. In addition, ASC-J9® could suppress AR-mediated tumor growth in several cancers, including liver cancer [38] and BCa [9]. Interestingly, other studies indicated ASC-J9® could also suppress prostate cancer progression via an AR-independent mechanism involving the suppression of STAT3-CCL2 signaling [11], which could explain why ASC-J9® could also suppress both BCa AR-positive TCC-SUP cells and AR-negative J82 cells.

Here we demonstrated that ASC-J9® could also alter NF-κB signals to increase BCa cell apoptosis and reduce cell proliferation. Our results proved that the level of phosphorylated NF-κB p65 protein (Ser536 and Ser529) was decreased by ASC-J9® treatment, suggesting that ASC-J9® could block the activities of kinases that are involved in NF-κB p65 phosphorylation. NF-κB is a key mediator of survival signaling via modulating the BCL-2 signals [39]. An early clinical study also suggested that NF-κB nuclear expression could be viewed as an independent prognostic indicator of adverse significance [40], and activation of NF-κB was linked to the development of platinum-resistance in BCa [41]. Another study also indicated that the dehydroxymethyl derivative of epoxyquinomicin C (DHMEQ), an NF-κB inhibitor, could induce apoptosis in advanced human BCa KU-19-19 cells [42], suggesting that NF-κB in BCa cells may represent a promising target to suppress BCa progression. Also, a recent study indicated that Cisplatin-induced apoptosis might arise through caspase-3-dependent pathways involving the nuclear IKK-α-mediated accumulation of p73α in response to Cisplatin [43].

There are several mechanisms responsible for development of Cisplatin-resistance. For example, the induced expression of drug resistance genes to reduce Cisplatin bioavailability within cells, the defects in the DNA repair mechanism, and the altered signals to prevent apoptosis following Cisplatin-induced DNA damage [44]. Several potential therapeutic approaches to overcome these molecular mechanisms to increase response rates and delay resistance have been proposed, including using antisense BCL-2 oligonucleotide to increase the cytotoxicity of Cisplatin [45] and targeting DNA repair capacity to enhance the sensitivity of cells to Cisplatin [46]. Zhang Q et al. proved that the miR34a/GOLPH3 axis abrogates BCa cells’ chemoresistance via reducing cancer stemness [47]. Our previous work also demonstrated a tumor suppressor gene, Maspin, enhanced Cisplatin chemosensitivity in BCa T24 and 5637 cells and correlated with prognosis of muscle-invasive BCa patients receiving Cisplatin based neoadjuvant chemotherapy [48]. However, few of these approaches have been translated into clinical applications. Our finding that NF-κB and AR signals were altered in miBCa, and targeting these two signals with ASC-J9® increased the sensitivity to and cytotoxicity of Cisplatin, may represent a new more effective therapy to suppress the miBCa.

In addition to Cisplatin, our results also demonstrated that ASC-J9® treatment could increase the efficacy of two other chemotherapy drugs, Doxorubicin and Mitomycin C, to suppress miBCa cell growth, suggesting that ASC-J9® might have a broader effect to increase the efficacy of some selective chemotherapy drugs. Combining these chemotherapy drugs with ASC-J9® to suppress miBCa during neoadjuvant chemotherapy (chemotherapy before surgery or radiation therapy) and adjuvant chemotherapy (chemotherapy after surgery or radiation therapy) may represent an interesting and important approach to improve patients’ survival in the near future. In this regard it is important to note that Doxorubicin is often used with Cisplatin and other agents in treating miBCa [5, 49]. Additionally, because intravesical instillation of Mitomycin C is used to treat nmiBCa, perhaps combining ASC-J9® with this treatment would improve its efficacy.

In the current study, we have demonstrated in vitro and in vivo that ASC-J9® could increase the efficacy of chemotherapy with Cisplatin to suppress BCa cell growth via altering apoptosis and proliferation. This conclusion is consistent with the TCGA database showing that the receptor tyrosine kinase (RTK)/RAS pathway, involved in cell cycle signaling, was altered in 44% of miBCa patients and that the TP53 tumor suppressor was functionally inactive in 76% of miBCa patients [8], suggesting that targeting cell growth with increasing cell apoptosis can better suppress the BCa progression.

A recent trial with Atezolizumab, an engineered humanized immunoglobulin G1 monoclonal antibody that binds selectively to the programmed death ligand 1 (PD-L1) in locally advanced or metastatic BCa patients who have progressed after platinum-based chemotherapy, found it had clinical benefits and good tolerability [50]. Nivolumab, a human IgG4 anti-PD-1 monoclonal antibody, also demonstrated clinical benefits [51], suggesting the great promise for immune checkpoint inhibitors in treating advanced BCa. Our finding that combined ASC-J9® and Cisplatin treatment could increase BCa cell apoptosis could be perceived as ‘non physiological’ by the immune system, which reacts by triggering an anti-tumor host immune defense [52]. Therefore, although the combination therapy of ASC-J9® and Cisplatin can induce cancer cells into the quiescent state, leading to remission, this chemotherapy in combination, alone and/or sequenced with immunotherapy, may lead to better therapeutic benefits because the apoptotic cells induced by anti-tumor agents could expose hidden tumor antigens to initiate immune responses.

## Conclusions

In conclusion, increased expression of NF-κB and AR was observed in miBCa. We demonstrated in vitro and in vivo that ASC-J9® could increase the efficacy of chemotherapy with Cisplatin to suppress BCa cell growth via altering the cell apoptosis and proliferation. ASC-J9® could be a promising chemotherapeutic sensitizer for miBCa.

## Additional files

Additional file 1: **Figure S1.** The illustration of xenografts and timeline of treatment regimen with Cisplatin (Cis) and ASC-J9® (ASC). Red arrows = ASC, black arrows = Cis. (PDF 84 kb)

Additional file 2: **Figure S2.** The representative images of apoptosis TUNEL assay in J82 (A) and TCC-SUP (B) cells. (PDF 504 kb)

## Data Availability

All data and materials used for/during this study are included in this published article.

## Abbreviations

AR: Androgen Receptor
BrdU: Bromodeoxyuridine
CI: Combination Index
Cis: Cisplatin
Dox: Doxorubicin
miBCa: Muscle Invasive Bladder Cancer
Mito: Mitomycin C
NF-κB: Nuclear Factor-kappa B
TMA: Tissue microarray
