# Supplementary figures and images for: ASC-J9® increases the bladder cancer chemotherapy efficacy via altering the androgen receptor (AR) and NF-κB survival signals
Source: J Exp Clin Cancer Res. 2019 Jun 24;38:275. doi: 10.1186/s13046-019-1258-0 (PMC6592003; doi:10.1186/s13046-019-1258-0)

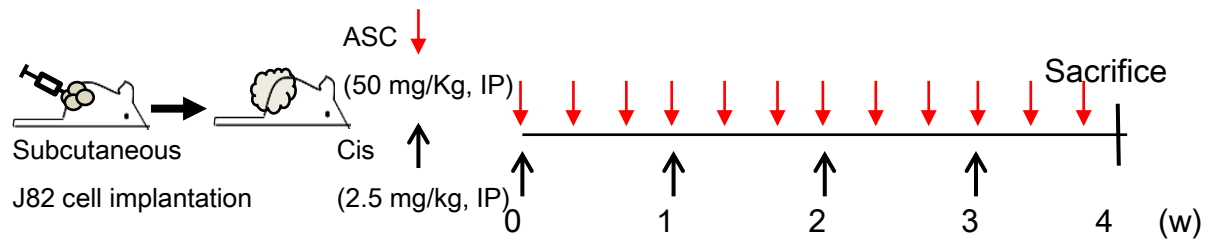

Supplement: Supplementary file 1 — Figure S1. The illustration of xenografts and timeline of treatment regimen with Cisplatin (Cis) and ASC-J9® (ASC). Red arrows = ASC, black arrows = Cis. (PDF 84 kb) [file 13046_2019_1258_MOESM1_ESM.pdf]

**A**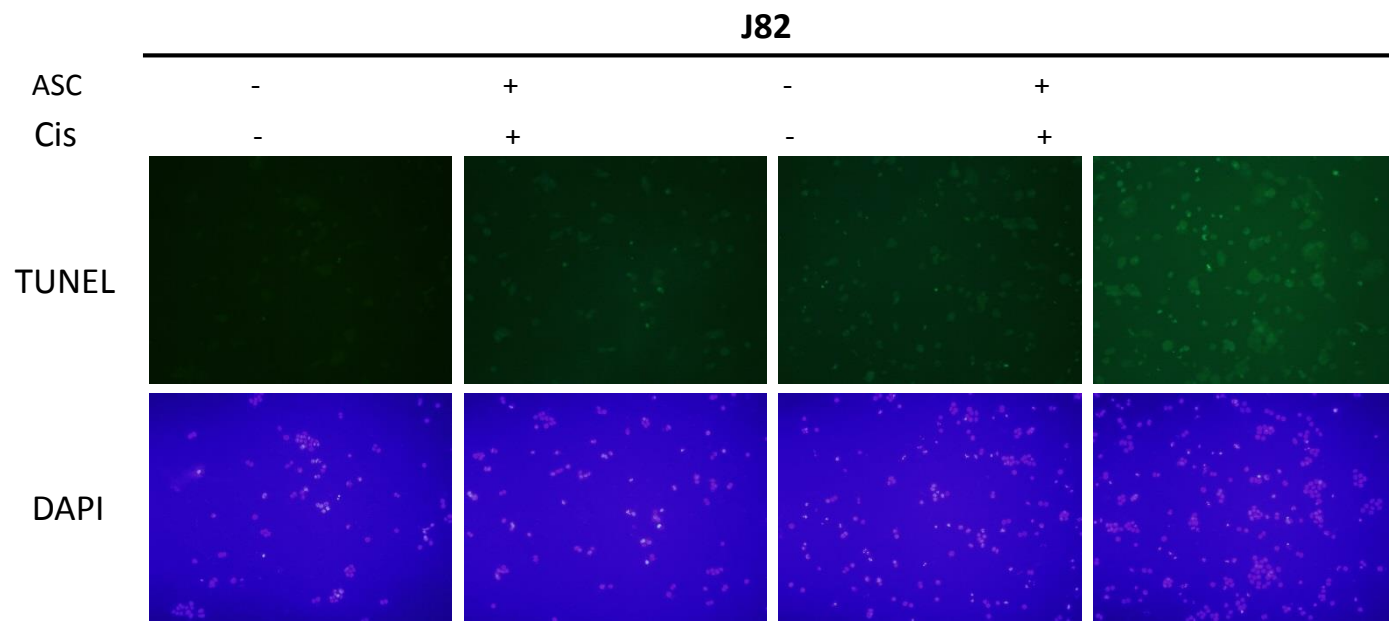**B**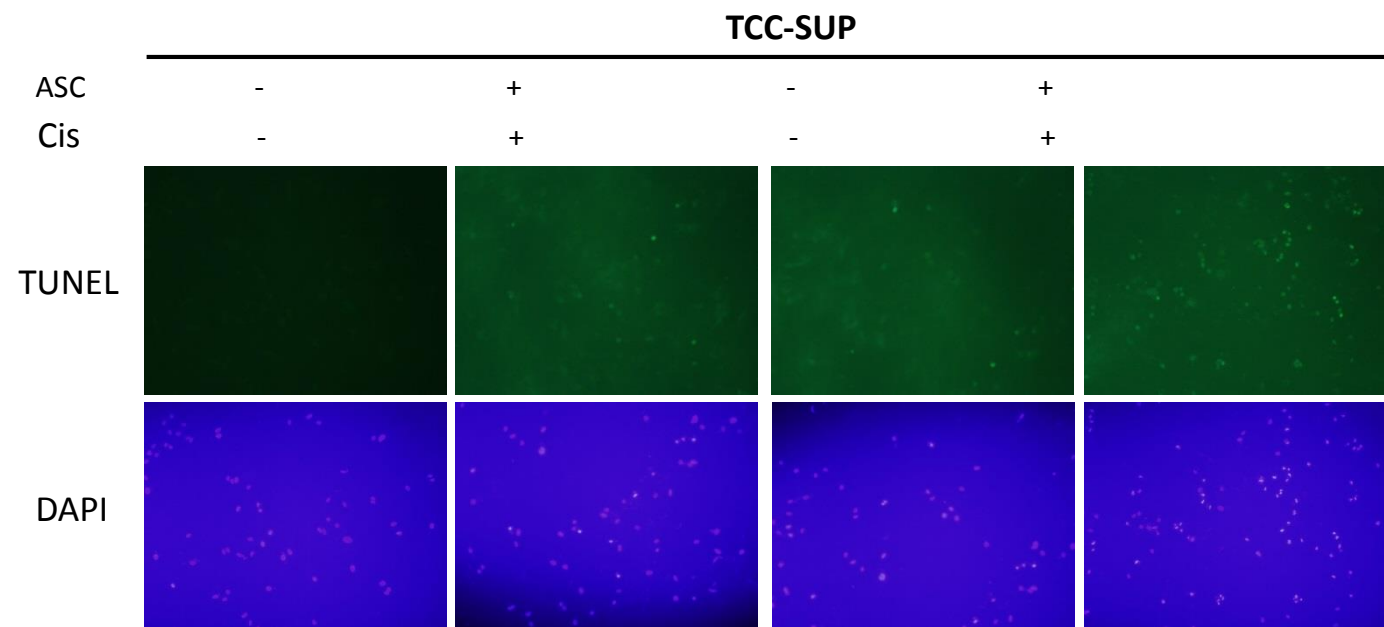

Supplement: Supplementary file 2 — Figure S2. The representative images of apoptosis TUNEL assay in J82 (A) and TCC-SUP (B) cells. (PDF 504 kb) [file 13046_2019_1258_MOESM2_ESM.pdf]
